# Orthopedic Telemedicine Outpatient Practice Diagnoses Set during the First COVID-19 Pandemic Lockdown—Individual Observation

**DOI:** 10.3390/ijerph19095418

**Published:** 2022-04-29

**Authors:** Wojciech Michał Glinkowski

**Affiliations:** 1 Center of Excellence “TeleOrto” for Telediagnostics and Treatment of Disorders and Injuries of the Locomotor System, Department of Medical Informatics and Telemedicine, Medical University of Warsaw, 00-581 Warsaw, Poland; w.glinkowski@gmail.com; 2 Polish Telemedicine and eHealth Society, 03-728 Warsaw, Poland; 3 Gabinet Lekarski, 03-728 Warsaw, Poland; 4 Centrum Medyczne PZU Zdrowie, 02-715 Warsaw, Poland

**Keywords:** COVID-19, telemedicine, orthopaedic, outpatient clinic, ICD-10, diagnosis, timing

## Abstract

The COVID-19 pandemic has caused a substantial intensification of the telemedicine transformation process in orthopedics since 2020. In the light of the legal regulations introduced in Poland, from the beginning of the SARS-CoV-2 pandemic, physicians, including orthopedic surgeons, have had the opportunity to conduct specialist teleconsultations. Teleconsultations increase epidemiological safety and significantly reduce the exposure of patients and medical staff to direct transmission of the viral vector and the spread of infections. The study aimed to describe diagnoses and clinical aspects of consecutive orthopedic teleconsultations (TC) during the pandemic lockdown. The diagnoses were set according to the International Classification of Diseases (ICD-10). Hybrid teleconsultations used smartphones and obligatory Electronic Health Record (EHR) with supplemental voice, SMS, MMS, Medical images, documents, and video conferencing if necessary. One hundred ninety-eight consecutive orthopedic teleconsultations were served for 615 women and 683 men (mean age 41.82 years ± 11.47 years). The most frequently diagnosed diseases were non-acute orthopedic disorders “M” (65.3%) and injuries “S” (26.3%). Back pain (M54) was the most frequent diagnosis (25.5%). Although virtual orthopedic consultation cannot replace an entire personal visit to a specialist orthopedic surgeon, in many cases, teleconsultation enables medical staff to continue to participate in providing medical services at a sufficiently high medical level to ensure patient and physician. The unified approach to TC diagnoses using ICD-10 or ICD-11 may improve further research on telemedicine-related orthopedics repeatability. Future research directions should address orthopedic teleconsultations’ practical aspects and highlight legal, organizational, and technological issues with their implementations.

## 1. Introduction

Telemedicine (TM) provides a safe and effective path to healthcare delivery [1,2,3]. The role of TM is rapidly evolving in various medical specialties, including orthopedics [4,5,6]. The coronavirus disease 2019 (COVID-19) pandemic has accelerated the shift towards remote consultations [6] in the medical field, including the musculoskeletal system. Remote consultation has entered primary and specialist healthcare since the onset of the COVID-19 pandemic to limit potential cross-contamination (staff-patient or patient-patient) [7]. Risks, barriers, but also facilitators that have emerged during the pandemic in relation to general and outpatient orthopedic TM have already been recognized internationally [8,9]. However, regardless, lockdowns have accelerated the implementation of TM in most countries around the world [3,10,11,12]. In orthopedics, TM was reported to be cost-effective and provided access to specialized care in various conditions [13,14,15,16]. American Academy of Orthopedic Surgeons [17], British Orthopaedic Association [18], and Australian Orthopaedic Association [19] have urgently advocated telemedicine during the COVID-19 pandemic to prevent disease transmission without hindering providing services to orthopedic patients. In a period of social isolation, the significant benefits of telemedicine have been recognized as an essential form of assistance in cases of infection for patients and healthcare professionals and to ensure continuity of healthcare while reducing the risk of spreading infectious diseases. Providing Teleconsultations (TC) has led to a significant reduction in the exposure of patients and medical staff to direct transmission of the viral vector and the spread of infectious diseases [20,21,22,23,24,25,26]. A study by Parisien et al. [17] demonstrated that academic orthopedic centers are taking new initiatives in telehealth, taking into account the needs of patients and the burden of COVID-19 disease. TM made it possible to provide medical care to quarantined patients and offer services to people exposed to infection with a virus of remarkably high virulence. The available and developed telemedicine technologies allow this time to change medical care and organization with the full approval of the healthcare regulations of all countries. Outpatient care and constant care of orthopedic patients during the coronavirus pandemic turn out to be difficult without a systematic workflow, proper coordination, and careful selection of patients for teleconsultation. Poland’s laws and regulations have increased a physician’s ability, including an orthopedic surgeon, to communicate virtually with the patient, practice, make decisions together, progress reports safely and effectively, and receive appropriate remuneration for the services provided. The legal basis is the Medical Activities Act (UoDzL), 96 the article 3, item 1, which states that “providing healthcare services” … “may be provided via teleinformatic or communications systems” [27].

Orthopedic TC requires specific skills in remote physical examination of patients [28,29,30,31]. The medical practitioner performing a virtual musculoskeletal examination should be familiar with specific physical examination techniques that the guided patient can self-perform while evaluating the shoulder, hip, knee, ankle, spine, or another part of the musculoskeletal system. An orthopedic surgeon should also be equipped with tools for reading photographs, videos, and medical images delivered by the patient to facilitate teleconsultation.

The study aimed to present a cross-section of diagnoses made in outpatients scheduled for orthopedic teleconsultation based on patients’ preferences in a subscription healthcare system during the first COVID-19 pandemic lockdown.

## 2. Materials and Methods

Due to the implementation of epidemic and lockdown regulations, the outpatient clinic was locked for orthopedic consultations in person, switching to smartphone teleconsultations. The analyzed data were collected from 23 March 2020 to 1 June 2020. The description intends to characterize patients and present diagnoses set due to consecutive orthopedic teleconsultations. This retrospective study describes the experiences of individual center. The study has a descriptive nature and intends to show a wide range of various problems that push patients to obtain medical care over telemedicine regardless of barriers to face-to-face communication. The study was not a medical experiment but was conducted based on medical records, without the active and passive participation of the people covered by the documentation. The tests did not require performing additional procedures and obtaining further information beyond the standard documentation of a given medical practice on the archival material in its possession. Orthopedic teleconsultations were delivered to the scheduled patients by the same orthopedic surgeon. The patients were informed about possible risks, privacy issues, and compliance with GDPR. Information security requirements were fully secured to prevent data loss. TCs were served only to patients who signed informed consent to use health services under these subscriptions. Using the scheduling application or the call center, the patient could reserve their own teleconsultation time and date. All teleconsultations planned and scheduled by the call center allowed for the improvement of patient identification. The principle of signing up for consultations was “first come-first served.” There were first visits and follow-up visits available. While conducting teleconsultations date and time of the call were recorded, the physician was identified (name, professional role, location, medical service provider), and the patient identity (or parent or legal guardian of the patient) was confirmed.

The patients could obtain their orthopedic teleconsultations (TC) over the smartphone. When necessary, the communication could be easily changed into a video consultation using a smartphone (simultaneous EHR, voice, image, documentation, and “Whats’up” videoconferences). Consultations were conducted for patients from several cities across Poland located in various Voivodeships (West Pomeranian, Lower Silesia, Opolskie, Silesian, Lesser Poland, Podkarpackie, Lodz, Mazovia, and Pomeranian). These consisted of simultaneous and obligatory EHR for patient documentation, voice, image, electronic documents, and videoconferencing. The dedicated mobile phone was used for exclusive images received over the MMS; the Electronic Health Record online system was linked. The call center supported the scheduling of patients. Video communication was used when the patient’s description of the signs and symptoms was inconclusive or misleading.

All necessary electronic documents, referrals, prescriptions, and certificates were incorporated into the EHR. The primary etiology of patients’ symptoms and findings during their TC were used to establish the initial or final diagnosis. The diagnoses were set according to the International Classification of Diseases (ICD-10). ICD-10 is the foundation for identifying worldwide health trends and statistics, the international standard for disease and health reporting, and the diagnostic classification for clinical and research purposes.

### Statistical Analysis

Groups and subgroups of patients and diagnoses were available for the analysis. The group was divided into age subgroups every five years except for patients under 14 and over 65. The results of the youngest and oldest patients were analyzed together. Descriptive statistics were calculated to characterize multiple variables. Spearman’s Rank Correlation was used to measure the correlation between two ranked variables and determine the association’s strength and direction between data sets. Pearson’s chi-square test of association was used to discover a relationship between categorical variables. One-way Kruskal–Wallis analysis of variance was used to explain which specific independent variable groups were statistically significantly different. A *p*-value < 0.05 was considered statistically significant.

## 3. Results

### 3.1. Descriptive Statistics

#### 3.1.1. Conducted Teleconsultations

The study included a group of 1298 consecutive orthopedic teleconsultations. Among the respondents, 615 were women—47.4%, and 683 were men—52.6%. The subjects were aged from 8 to 81. The mean age of the respondents was 41.82 years ± 11.47 years (median 41 years). A total of 1836 telephone calls were needed to complete all TCs. Almost 30% (588) of orthopedic surgeons’ calls were unanswered. Electronic Health Record was used for medical documentation paperwork. The average consultation time duration was 14 min and 30 s. 246 MMS and 962 SMS messages were exchanged with patients. Over two hundred patients referred to Medical Imaging returned their images over the individual PACS Server for supplemental evaluation.

#### 3.1.2. Diagnoses

Patients with various diagnoses were subjected to orthopedic teleconsultations (Table 1). In this study, patient’s diagnoses were made mostly within four groups of diseases and disorders, namely, diseases of the nervous system [ICD-10 chapter VI (G)]; diseases of the musculoskeletal system, and connective tissue [ICD-10 chapter XIII (M)]; and injury, poisoning and other inevitable consequences of external causes [ICD-10-chapter XIX (S and T)].

The diseases and disorders of the musculoskeletal system were more frequently diagnosed “M”-847 (65.3%) based on the ICD-10. Injuries were less frequent in the analyzed group “S”-341 (26.3%). The other diagnoses accounted for 8.5% of the total.

Back pain (M54) was the most frequent diagnosis (331 cases–25.5%). The cases with overuse of the musculoskeletal system (M70) were diagnosed in 144 cases (11.1%). Other orthopedic diseases (M) were also frequent (209 cases–16.1%). One hundred and eighty-nine patients (14.6%) were teleconsulted due to Musculoskeletal injuries (ICD–10 S group) (Table 2).

The most frequent main groups of diseases (ICD-10 Chapter diagnosis groups) consulted remotely during first, second, third, and fourth or later consultations are presented in the Table 3.The office work, particularly the home office during pandemic, could provoke mononeuropathies of the upper extremity; back and neck related disorders, knee injuries; The Back pain related (M43–M54), enthesopathy (M771; M77.3); shoulder disabilities (M75); and posttraumatic injuries (S) or posttraumatic disorders (T). Knee, ankle, and wrist injuries were most frequent (S83, S93, S63). Patients also complained about some remnants of the previous injuries to the upper and lower extremities (T92 and T93).

Disorders diagnosed during consecutive virtual consultations are presented in Table 4. The fourth and following TCs were aggregated into a single column. The percentages of cases diagnosed with G56 are calculated for all patients. Empty fields (#) of detailed diagnoses in the case of TC 1 mean that a more comprehensive initial diagnosis was made, and at the next visit, based on the assessment of the results of additional tests (i.e., imaging), a more detailed diagnosis could be made. The lack of precise diagnoses during the following TCs means that the patient’s primary diagnosis was different, usually due to the new musculoskeletal complaints.

Most of the patients suffered from a single medical problem (single diagnosis). Over 15% of patients complained about comorbidities (Table 5) or at least recalled them during the interview by presenting a medical record confirming diagnoses. Comorbidities were noted in the EHR system and considered necessary while prescribing medication.

The prevalence of particular groups of diseases in specific age groups presented in Table 6 was statistically significantly higher (Pearson’s chi-square value-χ^2^ (104) = 344.91; *p* < 0.001). The upper index stars mark the age group in which they occurred most often in each diagnosis.

Disease diagnoses in Table 7 varied according to the sex of the patients (Pearson’s chi-square value χ^2^(13) = 34.19; *p* = 0.001). The gender in which they occurred more frequently was marked with upper index stars for each diagnosis group.

The types of diagnoses differed depending on the age range of the patients (χ^2^(688) = 1807.24 *p* < 0.001). Disease diagnoses differed according to the sex of the patients (χ^2^(86) = 207.26 *p* < 0.001). The gender in which they occurred more frequently was marked in each diagnosis.

## 4. Discussion

The rapid adoption of telemedicine across various specialties and countries has shown promising results, with reported high satisfaction rates [32] due to its potential to provide quality care for patients with minimized risk of disease transmission. The reduced availability of specialized orthopedic care is making itself felt in society due to the pandemic, where many orthopedic patients are at substantial risk of severe disease from SARS-CoV-2. Rawal et al. [33] described a significant decrease in inpatient visits at the outpatient clinic due to the COVID–19 pandemics. The number of teleconsultations increased significantly due to the strictest regulations for social distancing. Scherer et al. [34] reported an increase in TM services from March to April 2020, 135% daily in acute cases and virtual health visits in response to COVID-19, from 94 per day to 4209 (4345%) in other cases.

The use of telemedicine in the health care system is based on legal regulations that were already established by legal acts before the pandemic. The Polish health care system reacted quickly and dynamically thanks to its complete preparation for providing remote medical services based on the teleinformatic systems. Since December 2015, TM in Poland is longer anymore understood as a medical experiment, thanks to previously signed legal acts [27,35]. It illustrates the remarkable healthcare transition into telemedicine. The patients can now be referred to a specialist, hospital, or diagnostics using e-Referrals available over the ICT system. Electronic tools available through the National e-Health Center in Poland enable e-Prescriptions.

Comparing the results described in this work (routine TCs) with those found in the literature (primarily occasional TCs) is not straightforward due to the difference in the frequencies of TCs. Lambrecht et al. [36] analyzed 410 orthopedic TCs performed by three orthopedic surgeons over two years averaging roughly 0.18 TC per day. Silva et al. [37] presented an observational, retrospective study that analyzed 1174 orthopedic asynchronous teleconsultations based on the large-scale public telehealth service. Their observation covered 2466 days in the years 2013–2020. Based on the data provided in the work of these authors and on the assumption that they conducted teleconsultations only on working days (Monday-Friday), they performed 0.66 TC per day or less if TC were provided 7 days a week. The authors stated that specialists teleconsulted in 48.7% of cases. Thus, the average number of consultations per day was 0.32 TC for a five-day working week. The authors did not specify whether they performed work on weekends. If this were the case, the average of TC performed would be even smaller. Chin et al. [4] carried out teleconsultations for 200 middle-aged women by 23 orthopedic surgeons from six subspecialties within ten months (from July 2020 to April 2021). That would make 0.66 TC per day and only 0.02 TC per surgeon per day. The recent report presents a relatively busy virtual orthopedic practice due to pandemics, with an average of 25.96 TC per day. It was challenging to compare the results and the conditions under which teleconsultation should be conducted. In pre-pandemic healthcare, it was clear that goodwill and a positive attitude toward telemedicine were necessary to implement them in reality. The conditions of the pandemic left patients and doctors no choice. In a situation where there was no other option, virtual consultations become an indispensable tool and environment for the doctor’s work.

The analyses of the distribution of patients’ diagnoses using teleconsultation are rarely published. Firstly, the studies conducted on an outpatient basis usually focus on patients’ relatively homogenous diagnosis groups [38,39,40,41,42,43,44,45]. Secondly, orthopedic teleconsultations are relatively new, leading researchers to closely scrutinize their feasibility, capabilities, and the satisfaction of patients and orthopedic surgeons. Thirdly, the organization of the outpatient clinics may be different in various countries. Initial musculoskeletal triage may be done with the primary care physician, who becomes responsible for referring the patient to an orthopedic specialist. A specialist can also perform Orthopedic TCs from the onset of MSK symptoms. In several countries and healthcare systems, general practitioners are usually responsible for the first-line evaluation and diagnostics of patients with musculoskeletal symptoms [6,46,47,48]. Choosing to visit a specialist over the subscription healthcare in Poland is a matter of patient choice. It does not have to be assigned by a family medicine physician or general practitioner (GP). It explains why there were a few primary diagnoses outside of orthopedics and traumatology in the studied group of patients. Patients suffering particular complaints may not know who the adequate specialist could be. In several cases, patients were confused by the detailed questions asked during the virtual interview concerning the anatomical location of the symptoms and appropriate description of symptoms.

It is surprising in the TM orthopedic literature review how simplified diagnoses are. Such descriptions alone force the readers to understand that an orthopedic telemedicine consultation may only be an inferior imitation of a consultation that can only occur face-to-face. Silva et al. found that complaints claimed for TC had been specified as bone in 30.2%, articular in 15.1%, and muscular in 7.6%. In the presented study, spine issues were virtually consulted in 25.5% of cases, similarly to Silva et al.’s study (23.9%). Only main groups of diagnoses and anatomical locations could be compared with other authors. They reported foot problems present in 16.6%, knee in 14.8%, and shoulder in 7.3% of patients. Chin et al. [4] described that gross of the orthopedic TC was focused on monitoring pain or discomfort or numbness; monitoring the range of motion of the affected joint; monitoring any change in functional status, or review of lab test results/imaging (e.g., X-ray, CT, MRI scans). The most frequent hand and reconstructive microsurgery patient groups were carpal tunnel syndrome and trigger finger or thumb. The readers can find more detailed diagnoses rarely. In this study, precise diagnoses were frequently set and expressed as IDC-10 codes consisting of letters, numerical codes, dots, and extension numbers. Typical shoulder and elbow cases were: frozen shoulder, tennis elbow, golfer’s elbow, and Osteoarthritis (OA) of the shoulder or elbow. The usual spinal cases consisted of neck or back pain due to cervical or lumbar radiculopathy. The hip and knee cases were OA of hip and knee, patellofemoral pain syndrome, and muscle strains. Virtual consultations of the foot and ankle area mainly dealt with ankle sprains, pes planus, and hallux valgus. Remote diagnosis of fractures is made successfully on an outpatient basis [49]. In the studies by Lambrecht et al. [36], diagnoses made during teleconsultation included fractures in 43% of patients, ligament injuries, joint swelling, or infection in 35% of cases, and dislocation in 4% of cases.

The analysis of teleconsulted cases shows that diagnoses from the M, S and T groups were significantly more frequent among males. Still, diagnoses from the G group were substantially more frequent among females. It is assumed that the period of the pandemic and the increase in online remote work “at home” may have increased the incidence of mononeuropathy and carpal tunnel syndrome. It seems typical for the study group, mostly aged around 40 years of age. Injuries and posttraumatic disorders are usually in the worldwide statistics more frequent in males. Mononeuropathies are also more frequent among females than typical epidemiology [50]. There was a trend towards a similar distribution of cases from the first TC to the next study group. Frequent disorders in general orthopedic practice cases indicate the need for specific examination skills to operate a TC. The remote examination skills of hand, knee, spine, and shoulder diseases are especially needed.

The effective treatment achieved remotely was already presented in the literature [29,30,31,45,51,52,53,54,55]. The treatment of undisplaced fractures, post-operative wounds [15,56], the injuries of the neck [45,51,52], wrist [57], knee [29,31,53], foot, and ankles [30,54,55] belong to the most frequently managed in orthopedic practice with telemedicine.

The barriers and limitations are frequently discussed issues of telemedicine [8,9]. The barriers are deepening disproportions in access to people’s services with limited access to telecommunications lines and digital devices [6,46,58]. Participating in a TC remains challenging for people who cannot cope with technology. Teleconsultation requires new understanding and skills from both the patient and healthcare professionals. Entering more and more precise areas of telemedicine, online consultations with a specialist will require new devices on the patient’s side, new skills to use them, and increasing the ability to read measurements from the tools used by patients [48,59]. Chin et al. [4] described that in the case of the presence of limiting factors (both on the part of the patient and the service provider), they introduced criteria for the precise selection of appointing patients suitable for teleconsultation. The authors considered that the face-to-face (F2F) consultation before discharge from the hospital or during an outpatient visit was an appropriate criterion for TC. The attending surgeon’s initial telephone interview could also be suitable for a TC. The lack of preselection in this study resulted in a specific need to solve various problems of the teleconsulted patient, including determining whether there are definite indications for F2F consultation.

The virtual examination has inherent limitations, and the orthopedist should evaluate when a face-to-face visit is necessary. A virtual orthopedic examination is becoming the most discussed topic in clinical orthopedic telemedicine. The physician must therefore acquire specific skills to verify the complaints of the patient during anamnesis as well during the virtually precise physical examination [28,29,30,31,53].

Tanaka et al. [28] have systematized the principles of physical examination for the proper functioning of virtual orthopedic consultations. An accurate implementation of telemedical orthopedic consultation, as in the case of other specialist virtual consultations, requires the appropriate preparation of both the patient and the physician.

Additional equipment can be suitable for TC enhancement, for example, the internet-based goniometer during the virtual examination [60].

There are several prerequisites to providing TC. To meet the inclusion criterion, the patient should be clinically stable, with no expected deterioration in health and functioning. Frequently, a TC is accepted after a physical examination during the F2F visit [4,28]. The condition for conducting the TC is to have logistic requirements for a telemedicine conference (laptop, webcam, application for videoconference, headphones/earbuds) and the ability to configure videoconference easily. TC participants should understand the nature, purpose, benefits, significant limitations, material risks, and alternatives of the TC services and agree to proceed. In the present study, the orthopedic center progressed without significant patient selection challenges without the preselection of cases.

Due to the pandemic circumstances, there was no alternative for TC in the study group; therefore, patients needed to participate in TC with the highest preference due to the limits of the other options. In addition, under such forced conditions, it was impossible to conduct a study comparing the remote group with the F2F of patients.

The patient should be logistically prepared for the teleconference (laptop, webcam, videoconference application, headphones/earbuds, and other necessary equipment), and it should be possible to set up the videoconference easily. The patient should understand the nature, purpose, benefits, significant limitations, and significant risks and alternatives of TC services and consent to the teleconsultation session. The telemedicine setting can be difficult for elderly orthopedic patients. However, telemedicine-oriented patient education may well solve the problem [37,61,62]. Studies conducted for virtual consultations for orthopedics usually focus on new solutions and technologies [59,63,64,65,66,67,68,69], patient and clinician satisfaction [4,24,26,46,49,70,71,72,73,74,75,76,77], clinical outcome measures [78,79,80,81,82], and cost analysis of traditional versus teleconsultation [82,83,84,85,86,87].

Teleconsultation remains frequent despite less stringent compliance with mask use and social distancing laws [4,59]. The increase in the use of telemedicine included both voice [88,89,90,91], multimedia [66,92,93,94,95,96], video consultations [34,63,74,97] as well as videoconferences [13,98,99,100,101,102,103,104,105]. Videoconferences ensure better patient identification and confirmation of the patient’s identity without additional difficulties [106,107]. However, phone calls remain the best understood for most patients [88,89,108,109]. Telephone teleconsultation remains an essential and recognized means of providing remote medical services. Raad et al. [90] observed that telephone clinics were practical and superior to traditional clinics for a specific set of patients during the pandemic. Estel et al. [110] recommended orthopedic telemedical consultation because of the high acceptance, objective benefits, and similarity of clinical results with F2F visits.

The concept of hybrid TC and enhanced virtual orthopedic consultations is not new [111]. Yang et al. [84] pointed out the use of smartphones for remote diagnosis and treatment. The consultation showed no significant differences in patient history, inspection, palpation, or active range of motion results. Providing teleconsultations leads to a substantial reduction in the exposure of patients and medical staff to direct transmission of the viral vector and the spread of infections [20,21,22,23,24,25,26]. Therefore, when the incidence increases during the next epidemic wave, the transition to using only teleconsultation is justified.

A referral is usually required for an appointment with an orthopedic surgery specialist in several countries. In Poland, a referral to an orthopedic clinic may be issued by a primary care physician (family physician) or another physician providing services under a valid agreement with the National Health Fund. However, in the system of health subscriptions in Poland, the choice of visiting a specialist is a matter of the patient’s preferences. It is not subject to regulation by a family medicine doctor or general practitioner (GP). It explains why there were a few primary diagnoses outside of orthopedics and traumatology in the studied group of patients. In other reports, general practitioners are usually responsible for the first-line evaluation and diagnostics of the patients with musculoskeletal symptoms [6,46,47,48].

Medical imaging diagnostics, including radiographs, are usually obtained at outside facilities after referring the patient. Usually, the physician refers the patient to the medical imaging department [112]. A feasible solution for acquiring medical images from patients is mandatory. The follow-up of the orthopedic patients may require periodic diagnostic medical imaging. Radiograph analysis and angle measurements comprise an essential mechanism in diagnosing, treating, planning, and evaluating the results of orthopedic surgery [113].

The orthopaedic surgeons obtained the unquestionable comfort of providing remote medical services depending on the time and technical possibilities. Note that orthopedic TCs or virtual orthopedic consultations are facilitated when the clinical evaluation has been performed or considered unnecessary. They play a valuable role when skilled orthopedic surgeons and patients cannot participate in the orthopedic physical examination in person.

The limitation of this study is that the study was conducted only in outpatient conditions of orthopedics and trauma surgery in the Polish subscription healthcare system. The presented work does not include a broad discussion of the barriers and limitations of telemedicine in orthopedics. On the one hand, the current research was not aimed at analyzing them; on the other hand, in many studies, these issues have already been discussed quite extensively [37,61,62,114,115]. Weaknesses of the presented research result from the individual material may show some bias. However, the nature and requirements of the TCs provided were consistent with the high standards of medical services, as far as conditions allowed. The TCs were served with the utmost care for the patients’ health.

The strong point of the research is the accurate documentation and the size of the analyzed group of patients.

## 5. Conclusions

The COVID-19 pandemic has caused a substantial intensification of the telemedicine transformation process in orthopedics since 2020. In the light of the legal regulations introduced in Poland, from the beginning of the SARS-CoV-2 pandemic, physicians, including orthopedic surgeons, have the opportunity to conduct teleconsultations. Running teleconsultations increases epidemiological safety and significantly reduces the exposure of patients and medical staff to direct transmission of the viral vector and the spread of infections. The most frequently diagnosed diseases were categorized based on the ICD-10 classification and belonged to the group orthopedic disorders “M” (65.3%) and injuries “S” (26.3%). Back pain (M54) was the most frequent diagnosis (25.5%), followed by musculoskeletal overuse (M70) (11.1%). The lack of preselection in the present study resulted in a specific need to solve various problems of the teleconsulted patient and determine whether there are absolute indications for face-to-face consultations. An accurate implementation of telemedical orthopedic consultation requires the proper preparation of both the patient and the physician with skills, equipment, and telecommunication.

Hybrid teleconsultation with smartphones and Electronic Health Record can work well. Introducing additional elements such as SMS, MMS, video conference, and transferring medical images to a physician’s dedicated secure diagnostic server are essential factors enhancing the quality of the TC.

Although virtual orthopedic consultation cannot replace an entire personal visit to a specialist orthopedic surgeon, TC enables medical staff to continue providing medical services at a sufficiently high medical level.

A unified approach to TC diagnoses using ICD-10 or ICD-11 may improve further research on telemedicine-related orthopedics repeatability. The current and future use of telemedicine should elaborate on new virtual orthopedic examination and management standards. Future research directions should address orthopedic teleconsultations’ practical aspects and highlight legal, organizational, and technological issues with their implementations.

## Figures and Tables

**Table 1:** The table shows the distribution of general and musculoskeletal diseases, disorders, and injuries diagnosed according to the ICD-10 chapters during all TCs.

Main Diagnosis Groups–ICD 10	Number	%
B07-Viral warts	2	0.2%
C43-Malignant melanoma	2	0.2%
D18-Hemangiomas of any location	4	0.3%
F52 Sexual dysfunction, not caused by organic disorder or disease	1	0.1%
G56-Mononeuropathies in upper limb	42	3.2%
H81-Disorders of the vestibular system	1	0.1%
I83-Varicose veins of the lower limbs	2	0.2%
K-Diseases of the digestive system	1	0.1%
L-Diseases of the skin and subcutaneous tissue	5	0.4%
M-Diseases of the bone and joint systems, muscles, and connective tissue	847	65.3%
R10 Pain in the abdominal and pelvic area R60-Edema, not elsewhere classified	4	0.3%
S-Injury, poisoning	341	26.3%
T-Injury, poisoning	34	2.6%
Z71-Persons contacting the health service for consultations and advice other than those classified elsewhere	1	0.1%
Other	11	0.8%
Total	1298	100.0%

**Table 2:** The table presents the most frequent diagnoses made in the study.

Most Frequent ICD-10 Diagnoses	Number of Cases	%
G56	42	3.2%
M54	331	25.5%
M65	34	2.6%
M70	144	11.1%
M77	54	4.2%
S13	27	2.1%
S63	21	1.6%
S80	17	1.3%
S93	87	6.7%
Other M	209	16.1%
M75	75	5.8%
S	189	14.6%
T	34	2.6%
Other and Unspecified	34	2.6%
Total	1298	100.0%

**Table 3:** The most frequent main groups of diseases consulted remotely during first, second, third, and fourth or later consultations.

Teleconsultation Number (TC)	Overall Patients Number	G	M	S	T
1	695	25 (3.59%)	466 (67.05%)	170 (24.46%)	20 (2.87%)
2	282	9 (3.19%)	174 (61.70%)	83 (29.43%)	11 (3.9%)
3	150	4 (2.66%)	93 (62.0%)	45 (30.0%)	3 (2.0%)
4 and later	171	4 (2.33%)	116 (67.83%)	43 (25.14%)	1 (0.58%)

**Table 4:** Percentage of cases with specific diagnoses during consecutive TCs.

ICD-10 Diagnosis	Disorder Description	First TC	Second TC	Third TC	Fourth and Following TC
G56	Carpal tunnel syndrome	25 (3.59% of all cases)	9 (3.19% of all cases)	4 (2.66% of all cases)	4 (2.33% of all cases)
M23	Chronic knee disorders	(0)	14 (8.04%)	9 (9.67%)	M23-8 (6.89%)
M43–M54	Spine pain related problems	186 (39.91%)	63 (36.2%)	35 (37.63%)	M54-65 (56.03%)
M65	Articular inflammation/infection	(0)	(0)	(0)	7 (6.03%)
M70–M77	Musculoskeletal overuse disorders and enthesopathy	163 (34.97%)	43 (24.69%)	17 (18.27%)	16 (13.78%)
M75.8–M75.9	Shoulder related problems	35 (7.51%)	18 (10.34%)	12 (12.9%)	6 (5.17%)
M94	Articular cartilage disorders	(#)	6 (3.44%)		4 (3.44%)
S13	Neck Injuries	(#)	6 (7.22%)	(#)	10 (23.25%)
S63	Wrist injuries	10 (5.88%)	6 (7.22%)	4 (8.88%)	(0)
S83–S83.6	Knee injuries	36 (21.17%)	21 (25.3%)	10 (22.22%)	(0)
S93–S93.6	Foot and ankle joint injuries	40 (23.52%)	23 (27.71%)	17 (37.77%)	20 (46.5%)
T92	Posttraumatic disorders of the upper extremity	10 (50%)	7 (63.63%)	(0)	(0)
T93	Posttraumatic disorders of the lower extremity	9(45%)	(0)	(0)	(0)

**Table 5:** The number of diagnoses (a single patient diagnosed with two or more diseases).

Number of ICD-10 Diagnoses	N	%
Single diagnosis	1062	83.6%
Two diagnoses	173	13.6%
Three diagnoses	21	1.7%
Four diagnoses	10	0.8%
More diagnoses	5	0.4%
Total	1298	100.0%

**Table 6:** The table presents the relationships between the most frequent diagnoses and a specific age range. * Upper index stars reflect the significance (*p* = 0.001).

ICD-10	08–14 (%)	15–20 (%)	20–25 (%)	26–30 (%)	31–40 (%)	41–50 (%)	51–60 (%)	61–65 (%)	66 and Older (%)
M (%)	6 (50%)	2 (40%)	36 (59%)	* 93 (75.0%)	* 253 (60.4%)	* 248 (66.7%)	* 138 (66.7%)	* 66 (84%)	* 8 (66.7%)
G (%)	0 (0%)	0 (0%)	1 (2%)	0 (0.0%)	* 10 (2.4%)	* 10 (2.7%)	* 18 (8.7%)	* 3 (4%)	0 (0.0%)
T (%)	* 1 (8%)	0 (0%)	1 (2%)	4 (3.2%)	14 (3.3%)	9 (2.4%)	5 (2.4%)	0 (0%)	* 1 (8.3%)
S (%)	* 3 (25%)	* 3 (60%)	* 22 (36%)	* 25 (20.2%)	* 131 (31.3%)	* 103 (27.7%)	41 (19.8%)	10 (13%)	3 (25.0%)

**Table 7:** The main types of most frequent diagnoses according to gender. * Upper index stars reflect the significance (*p* = 0.001).

ICD-10	Males	Females	Total
M (%)	448 (66.2%) *	402 (65.5%)	850 (65.8%)
G (%)	11 (1.6%)	31 (5.0%) *	42 (3.3%)
T (%)	24 (3.5%) *	11 (1.8%)	35 (2.7%)
S (%)	184 (27.2%) *	157 (25.6%)	341 (26.4%)

## Data Availability

Data supporting reported results can be available on-demand for the readers.
